# Genetic biomarker prediction based on gender disparity in asthma throughout machine learning

**DOI:** 10.3389/fmed.2024.1397746

**Published:** 2024-09-13

**Authors:** Cai Chen, Fenglong Yuan, Xiangwei Meng, Fulai Peng, Xuekun Shao, Cheng Wang, Yang Shen, Haitao Du, Danyang Lv, Ningling Zhang, Xiuli Wang, Tao Wang, Ping Wang

**Affiliations:** ^1^Shandong Institute of Advanced Technology, Chinese Academy of Sciences, Jinan, China; ^2^Department of Pulmonary and Critical Care Medicine, Yantai Yeda Hospital, Yantai, China; ^3^Biomedical Engineering Institute, School of Control Science and Engineering, Shandong University, Jinan, China; ^4^School of Pharmacy, Shandong University of Traditional Chinese Medicine, Jinan, China; ^5^Shandong Academy of Chinese Medicine, Jinan, China; ^6^Tongji Medical College, Huazhong University of Science and Technology, Wuhan, China; ^7^Neck-Shoulder and Lumbocrural Pain Hospital of Shandong First Medical University, Jinan, China

**Keywords:** asthma, gender disparity, machine learning, biomarker, prevalence

## Abstract

**Background:**

Asthma is a chronic respiratory condition affecting populations worldwide, with prevalence ranging from 1–18% across different nations. Gender differences in asthma prevalence have attracted much attention.

**Purpose:**

The aim of this study was to investigate biomarkers of gender differences in asthma prevalence based on machine learning.

**Method:**

The data came from the gene expression omnibus database (GSE69683, GSE76262, and GSE41863), which involved in a number of 575 individuals, including 240 males and 335 females. Theses samples were divided into male group and female group, respectively. Grid search and cross-validation were employed to adjust model parameters for support vector machine, random forest, decision tree and logistic regression model. Accuracy, precision, recall, and F_1_ score were used to evaluate the performance of the models during the training process. After model optimization, four machine learning models were utilized to predict biomarkers of sex differences in asthma. In order to validate the accuracy of our results, we performed Wilcoxon tests on the genes expression.

**Result:**

In datasets GSE76262 and GSE69683, support vector machine, random forest, logistic regression, and decision tree all achieve 100% accuracy, precision, recall, and F_1_ score. Our findings reveal that XIST serves as a common biomarker among the three samples, comprising a total of 575 individuals, with higher expression levels in females compared to males (*p* < 0.01).

**Conclusion:**

XIST serves as a genetic biomarker for gender differences in the prevalence of asthma.

## 1 Introduction

Asthma is a chronic respiratory condition affecting populations worldwide, with prevalence ranging from 1–18% across different nations (1). This ailment is characterized by diverse respiratory symptoms and variable airflow limitation. Asthma represents a complex interplay between genetic and environmental factors, giving rise to a heterogeneous spectrum of clinical manifestations, airway inflammation, and remodeling (2). Presently, there is compelling evidence linking asthma to various inflammatory pathways (3), suggesting that this condition is not solely a straightforward, monocausal disease but rather a multifaceted and diverse syndrome with an array of inflammatory mechanisms (4).

The overall prevalence of asthma was estimated to be 4.2% (95% CI: 3.1–5.6) in a sample of 45.7 million Chinese adults. Among children, boys exhibit a higher asthma prevalence compared to girls; however, in women, the prevalence is approximately 20% higher than in men (5). Notably, this discrepancy may change during puberty. The higher prevalence in boys compared to younger girls can be partially attributed to the relatively smaller size of their airways in comparison to their lungs. A prospective study involving 19-year-old children revealed that 21% of those diagnosed with asthma at the age of 7 experienced resolution, 38% had recurrent asthma, and 41% had persistent asthma. Remission was more frequent among boys, but less noticeable in girls and patients with severe asthma or sensitivity to fur animals (6).

Despite the crucial role played by environmental factors in asthma development, genetic factors have also been identified as key contributors. Studies investigating the heritability of asthma (the extent of population phenotypic variation attributed to genetic variation among individuals within the population) have estimated it to range from 35 to 95% (7). Dogs and cats are the most prevalent domestic pets, and individuals with anaphylactic responses may experience significant asthma-related morbidity due to exposure to allergens from these animals (8). Approximately 25 to 65% of children with persistent asthma display sensitivity to these allergens (9, 10).

Research has confirmed that the severity of asthma and its diverse clinical phenotypes may be linked to specific pathogenic moleculars, identified as the asthma biomarkers (11). Elevated levels of type 2 cytokines such as IL-5, IL-4, IL-13, IL-25, IL-33, periostin, dipeptidyl peptidase-4, osteopontin, fractional exhaled nitric oxide, bromotyrosine, prostaglandin D2 and leukotriene E4, and thymic stromal lymphopoietin (TSLP) are emblematic biomarkers for the detection and diagnosis of T2-high asthma; conversely, for the diagnosis and monitoring of low T2 type asthma, only a limited number of available biomarkers are mediated by Th1 and Th17 cells, including TNF-α, IL-1β, IL-6, IL-8, IL-17, folliculin, S100A9, myeloperoxidase, neutrophil elastase, and brain-derived neutrophil factor (12). Moreover, asthma biomarkers are often closely associated with genetic factors, encompassing genetics, epigenetics, and transcriptomic studies (13). In light of these factors, the application of machine learning and artificial intelligence technologies will enhance the precision in identifying biomarkers for different asthma phenotypes.

Machine learning is a crucial branch of artificial intelligence, with its core focus on enabling algorithms to self-optimize through training datasets, thereby making predictions or decisions on unseen data (14). Machine learning and artificial intelligence have been widely applied in the medical field, such as in image recognition, intelligent diagnostics, healthcare, and biomarker prediction (15, 16). Ding et al. (17) explored asthma-related lipid metabolism-associated biomarkers in mouse samples through five types of machine learning models, ultimately identifying cholesterol 25-hydroxylase (CH25H) as a central lipid metabolic gene in asthma. Lin et al. (18) based on weighted gene co-expression network analysis and machine learning, found 11 hub genes from the GSE135192 data set that could serve as novel diagnostic markers and therapeutic targets for pediatric asthma. Camiolo et al. (19) performed machine learning classification of bronchial epithelial cell gene expression data and found that L18R1 (IL-18 receptor 1) was inversely associated with lung function and was highly expressed in the most severely asthmatic population.

Gender differences are another reason for asthma attacks. Asthma prevalence rises in boys during childhood. In contrast, the prevalence and severity of asthma increases as women become older. Gender differences in asthma prevalence have attracted widespread attention. In this study, we used machine learning to explore potential biomarkers.

## 2 Method

**Figure 1:**
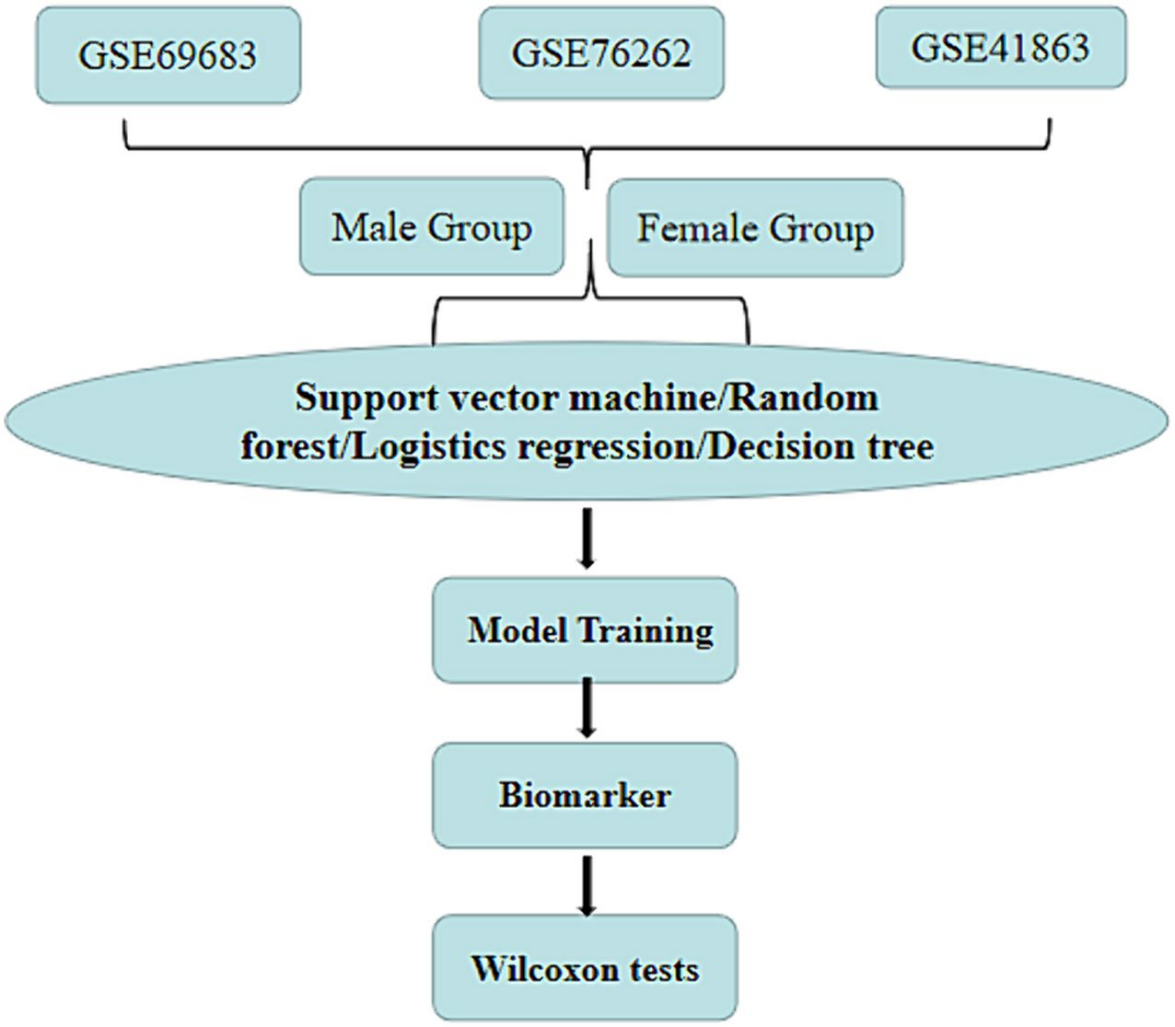
The flowchart of the research.

The process of this study is depicted in Figure 1. Firstly, we selectively extract three samples (GSE69683, GSE76262, and GSE41863) from the gene expression omnibus (GEO) database and categorize them into male and female groups based on gender. The data came from the gene expression omnibus database^1^ (20), which is a gene expression public database created in 2000 and contains high-throughput gene expression data around the world (21). Subsequently, we optimized the parameters of four machine learning models: support vector machine, random forest, logistic regression, and decision tree. We then input the optimized parameters into the machine learning models to predict biomarkers of gender-specific difference associated with asthma prevalence. Lastly, we validate our findings through the Wilcoxon test.

### 2.1 Data source

Data were obtained from three samples including No. GSE69683 (22), No. GSE76262 (23), and No. GSE41863 (24), in which we divided asthma patients into male group and female groups, involving a number of 575 individuals, including 240 males and 335 females (Table 1). Data set about GSE41863, GSE69683, and GSE76262 was obtained from sputum cells, blood sample and induces sputum, respectively. Subjects in GSE69683 were divided into severe, moderate, and healthy group according to grade of severity. Severe and moderate asthma subjects were merged, and divided into male and female group.

**Table 1:** Gender distribution in the sample.

Datasets	Female	Male	Age
GSE69683	243	170	≥27
GSE76262	70	47	—
GSE41863	22	23	—

### 2.2 Machine learning

Grid search and cross-validation were used to adjust model parameters for support vector machine, random forest, decision tree and logistic regression model. Parameter settings are shown in Table 2. For support vector machine model, kernel was setting as linear, and penalty coefficient was setting from 0.0005 to 100. N_estimators and Max_depth of random forest were from 10 to 500, and from 1 to 70, respectively. As for logistic regression model, *C* was setting from 0.001 to 11. Accuracy, precision, recall, and F_1_ score were used to evaluate the classification performance of the models during the machine learning process. As depicted in Table 3, TP represents the number of correctly classified positive samples, TN represents the number of correctly classified negative samples, FP represents the number of samples falsely classified as negative, and FN represents the number of positive samples incorrectly classified. All the aforementioned operations were carried out in Python3.7 software.

**Table 2:** Parameter settings based on grid search for model optimization.

Model	Parameters	Setting
Support vector machine	Kernel	Linear
	*C*	0.0005, 0.001, 0.005, 0.01, 0.05, 0.1, 0.5, 1, 5, 10, 20, 100
	Gamma	100, 50, 40, 30, 20, 15, 11, 9, 5, 7, 3, 1, 0.1, 0.01, 0.001
Random forest	N_estimators	10, 20, 30, 40, 50, 60, 70, 80, 90, 100, 150, 200, 300, 400, 500
	Max_depth	1, 2, 3, 4, 5, 6, 7, 8, 9, 10, 15, 20, 25, 30, 35, 40, 45, 50, 55, 60, 70, 80, 90, 100
Logistics regression	*C*	0.001, 0.003, 0.005, 0.007, 0.009, 0.1, 0.3, 0.5, 0.7, 0.9, 1, 3, 5, 7, 9, 11
Decision tree	Criterion	Gini, Entropy
	Max_depth	1, 3, 5, 7, 9, 15, 20, 25, 30, 35, 40, 50, 100, 200
	Max_leaf_nodes	1, 3, 5, 7, 9, 11, 15, 20, 30, 40, 50, 100

**Table 3:** Evaluating indicators.

Evaluation index	Function definition
Recall	$\text{Recall}=\frac{TP}{TP+FN}*100\%$
Specificity	$\text{Specificity}=\frac{TN}{TN+FP}*100\%$
Precision	$\text{Precision}=\frac{TP}{TP+FP}$
F_1_-score	$F_{1}\text{score}=2\frac{\text{Precision}*\text{Recall}}{\text{Precision}+\text{Recall}}$
Accuracy	$\text{Accuracy}=\frac{TP+TN}{TP+TN+FP+FN}*100\%$

### 2.3 Statistical analysis

In order to validate the accuracy of our results, we performed Wilcoxon test on the genes from the GSE69683, GSE76262, and GSE41863. The Wilcoxon test was operated in the website https://www.home-for-researchers.com/#/.

## 3 Result

### 3.1 Model training

The parameter optimization results for support vector machine, random forest, logistic regression, and decision tree using the grid search-cross validation method are shown in Table 4. For all three samples, the optimal parameters for support vector machine are *C* = 0.005, Gamma = 100, and kernel = linear. For sample GSE69683, the optimal parameters for random forest are Max_depth = 2 and N_estimators = 150. For sample GSE76262, the optimal parameters are Max_depth = 4 and N_estimators = 300. Lastly, for sample GSE41863, the optimal parameters are Max_depth = 90 and N_estimators = 20.

**Table 4:** The optimal parameters for the four models.

Model	Support vector machine			Random forests		Decision tree			Logistic regression
	*C*	Gamma	Kernel	Max_depth	N_estimators	Criterion	Max_depth	Max_leaf_nodes	*C*
GSE69683	0.0005	100	Linear	2	150	Gini	1	3	0.001
GSE76262	0.0005	100	Linear	4	300	Gini	1	3	0.1
GSE41863	0.0005	100	Linear	90	20	Gini	5	20	0.003

**Figure 2:**
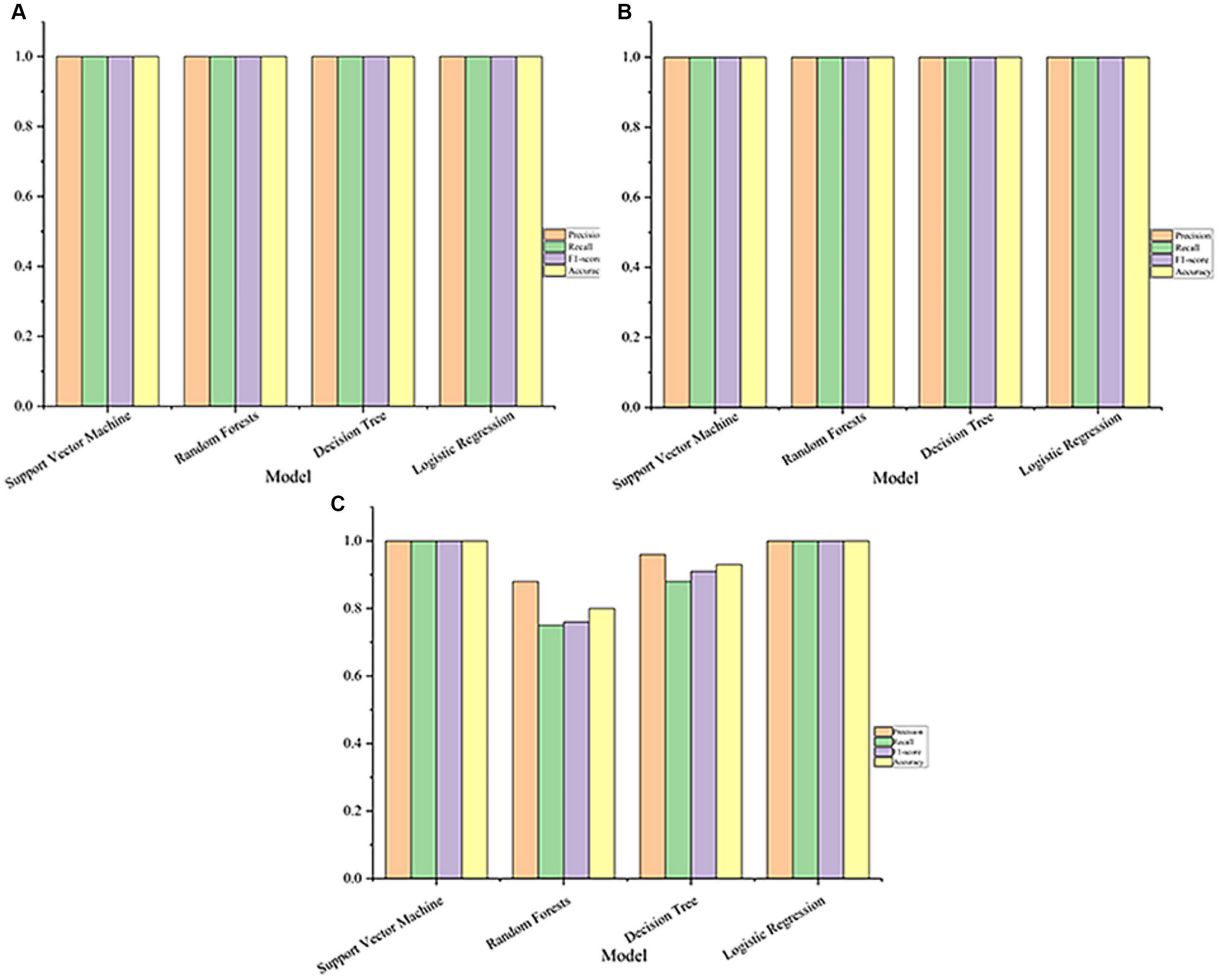
The performance of the machine learning model on the dataset (**A**, GSE76262; **B**, GSE69683; **C**, GSE41863).

The performance of each model with the optimal parameters obtained during training is shown in Figure 2. In datasets GSE76262 and GSE69683, support vector machine, random forest, logistic regression, and decision tree all achieve 100% accuracy, precision, recall, and F_1_ score, described in Figures 2A,B. However, in the dataset GSE41863, the random forest achieved an accuracy of 88%, a recall rate of 75%, an F_1_ score of 76%, and a precision of 80% (Figure 2C).

### 3.2 Biomarker prediction

Table 5 presents the intersection of the top 20 important genes in the feature ranking among four models when the model reaches its optimum. During blood sample GSE69683, support vector machine, random forest, logistic regression, and decision tree all ranked X-inactive specific transcript (XIST) among the top 20 genes. The intersection of support vector machine, random forest, and logistic regression models comprises TSIX, TTTY10, TTTY14, TTTY15, TXLNGY, USP9Y, UTY, and ZFY genes (Table 6) in blood sample GSE69683.

**Table 5:** Intersection of the top 20 genes ranked by feature importance among four models.

Model	GSE69683	GSE76262	GSE41863
Support vector machine Random forests Decision tree Logistic regression	XIST	TSIX XIST	TXLNGY USP9Y UTY XIST ZFY

**Table 6:** Intersection of the top 20 genes ranked by feature importance among three models.

Model	GSE76262	GSE69683	GSE41863
Support vector machine Random forests Logistic regression	DDX3Y EIF1AY KDM5D RPS4Y1	TSIX TTTY10 TTTY14 TTTY15 TXLNGY USP9Y UTY ZFY	TSIXT TTY15
Random forests Decision tree Logistic regression	TXLNGY USP9Y	Support vector machine Random forests Decision tree	ZNF107 ZNF471

The intersection of support vector machine, random forest, decision tree, and logistic regression models induces sputum sample GSE76262 are TSIX and XIST. The intersection of support vector machine, random forest, and logistic regression models consists of DDX3Y, EIF1AY, KDM5D, and RPS4Y1 genes in GSE76262.

In sputum cell sample GSE41863, the intersecting genes ranked among the top 20 by all four models are TXLNGY, USP9Y, UTY, XIST, and ZFY. The intersection of support vector machine, random forests, and logistic regression models includes TSIXT and TTY15.

**Figure 3:**
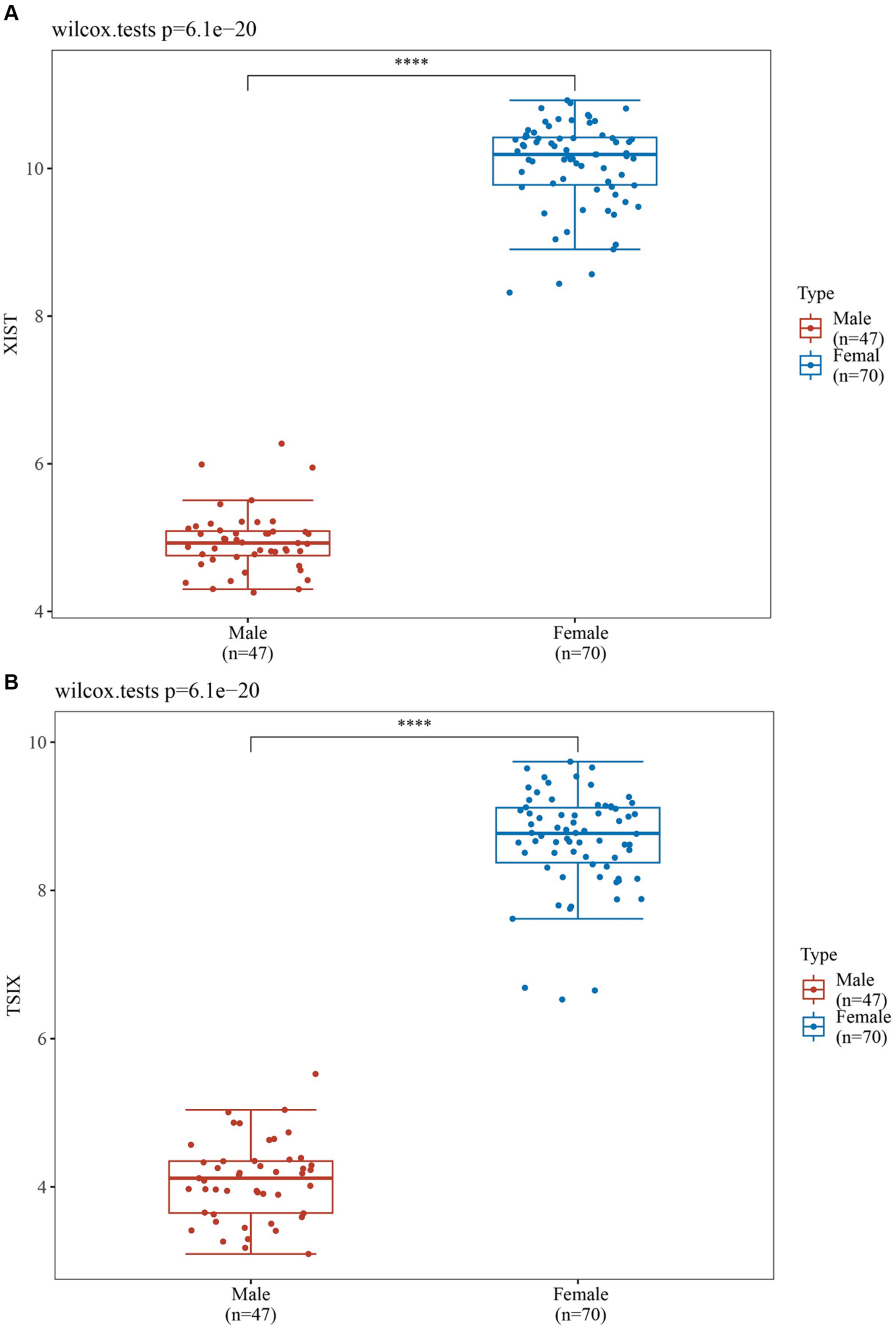
Results of Wilcoxon tests on GSE76262 (**A**, XIST; **B**, TSIX).

**Figure 4:**
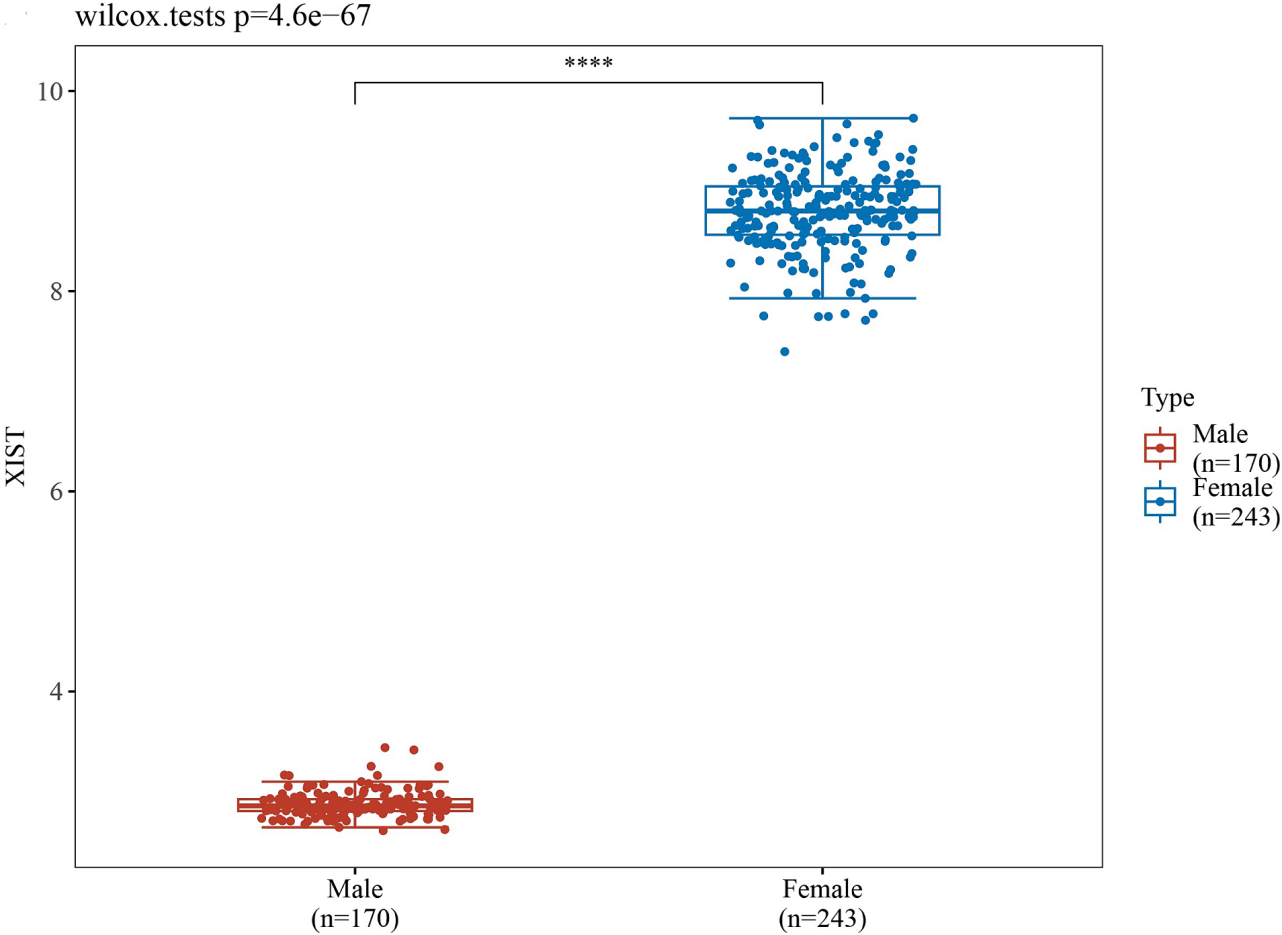
Results of Wilcoxon tests on GSE69683.

**Figure 5:**
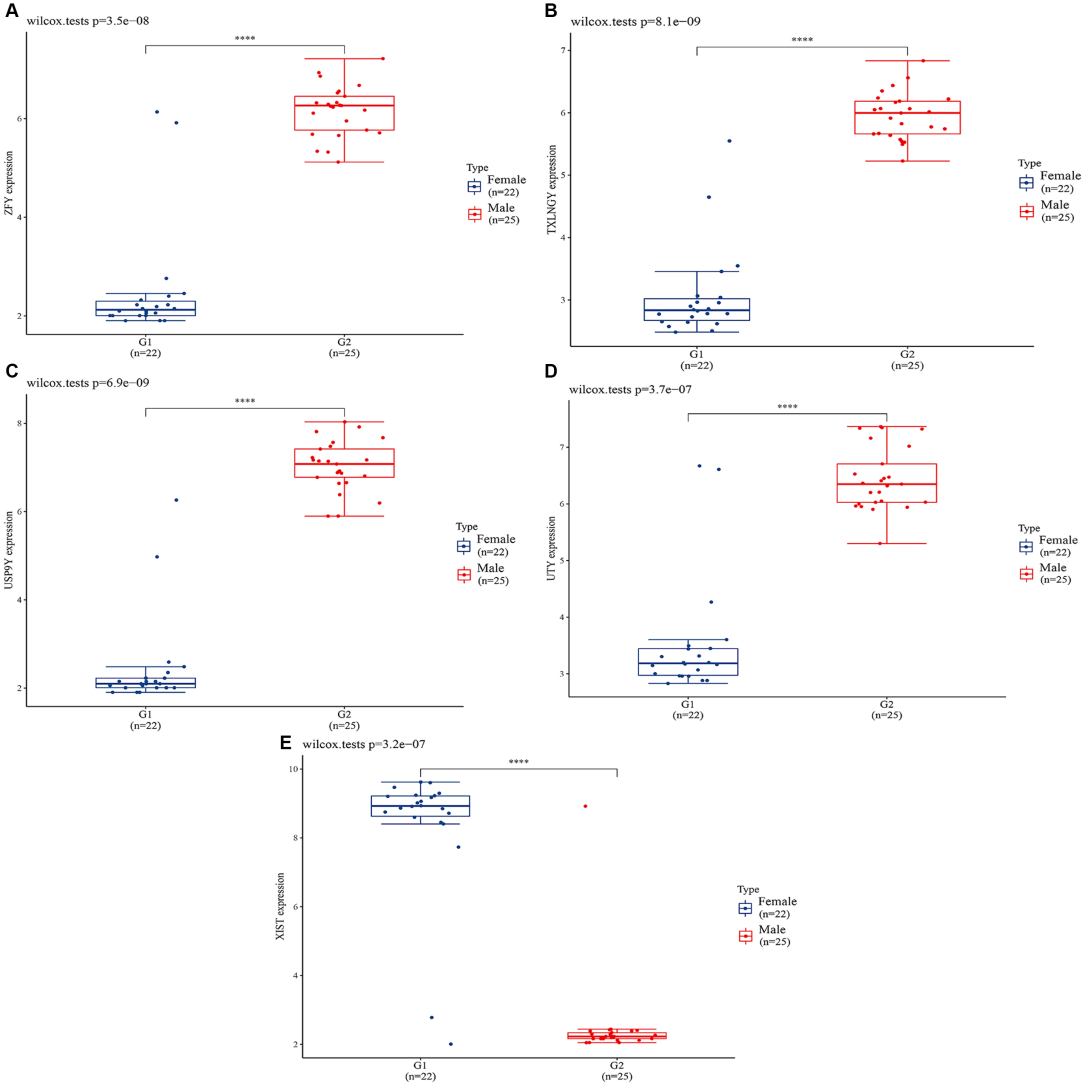
Results of Wilcoxon tests on GSE41863 (**A**, ZFY; **B**, TXLNGY; **C**, USP9Y; **D**, UTY; **E**, XIST).

In order to validate the accuracy of our results, we performed Wilcoxon tests on the genes from the GSE69683 (XIST), GSE76262 (TSIX and XIST) and GSE41863 (TXLNGY, USP9Y, UTY, XIST, and ZFY). As depicted in Figure 3, within the GSE 76262 dataset, there were 47 males (represented by the blue color) and 70 females (represented by the red color). TSIX and XIST exhibited higher expression in females and lower expression in males, with statistical significance (*p* < 0.001). The same result about XIST is observed in the GSE69683 and GSE41863 data sets, as illustrated in Figures 4, 5E. As shown in Figure 5, the expression of TXLNGY, USP9Y, UTY, and ZFY is significantly higher in males compared to females, with statistical significance (p < 0.001).

## 4 Discussion

Asthma is a common chronic inflammatory disease of the airways, characterized by variable and recurrent symptoms, reversible airflow obstruction, and bronchospasm (25). The etiology of asthma is complex and likely involves the interaction between genetic factors and environmental factors that are not fully understood yet. This study, based on machine learning, was purposed to investigate the genetic biomarkers that caused sex differences in asthma.

The gender disparity in the incidence of asthma has attracted considerable attention among scholars. The physiological variances in pulmonary development and structure may contribute to this phenomenon. Sex differences in lung development between males and females begin as early as weeks 16–24 of gestation (26). Female fetuses have smaller airways and a lower number of respiratory bronchioles compared to males; however, they exhibit a faster rate of maturation (27). Upon reaching adulthood, males and females are exposed to potentially distinct occupational and familial triggering factors that may influence asthma. Females have a greater opportunity to utilize cleaning agents within their domestic environment compared to males (28). Certain chemical substances present in these cleaning agents have the potential to induce respiratory allergic reactions or inflammation, subsequently leading to the onset of asthma.

The number of genes associated with the X chromosome was thought to influence the immune response and the development of autoimmune diseases, such as asthma. Taking toll-like receptor (an X-linked gene involved in innate immunity) as an example, TLR7-mediated HLADR + CD3–CD19-cell production of IFN-α was significantly upregulated in healthy women compared to healthy men. This suggests that the presence of two X chromosomes plays an important role in enhancing innate and adaptive immune responses (29). TLR7 could be capable of escaping X-chromosome inactivation in female immune cells, similar to TLR8, which also could evade X-chromosome inactivation in human monocytes and CD4 T cells. The co-dependent transcription of the active X chromosome and the escape from X-chromosome inactivation (XCI) both lead to higher protein abundance of TLR8 in female cells, which may impact the response to viruses and bacteria, as well as influence the risk of developing inflammation and autoimmune diseases (30).

The X-inactive-specific transcript (XIST) gene serves as a primary regulatory factor for X chromosome inactivation in mammals. In this study whether it’s a blood sample, an induced sputum sample, or a sputum cell sample, XIST ranked at the top of all four machine learning models in our predictions. XIST produces a long non-coding (lnc) RNA that accumulates throughout the entire length of the transcribed chromosome, recruiting factors to modify the potential chromatin and silence X-linked genes in cis. Previous studies have established a significant correlation between XIST and lung pathologies. In the context of lung cancer, Li et al. (31) discovered that XIST in metastatic non-small cell lung cancer (NSCLC) tissues facilitates TGF-β-induced EMT, as well as cell invasion and metastasis, through modulation of the miR-367/miR-141-ZEB2 axis. Additionally, XIST expression is elevated in response to the nicotine derivative nitrosamine ketone (NNK) in lung injury, influencing the aberrant expression of miR-328-3p (32). Furthermore, XIST plays a role in acute lung injury (ALI), Li et al. (33) observed upregulation of XIST in a lipopolysaccharide (LPS)-ALI mouse model and in lung endothelial cells; knockdown of XIST inhibited the LPS-induced inflammatory response and apoptosis in these cells. While numerous studies have substantiated the association between XIST and various lung diseases, its relationship with asthma has been less explored. In the present study, we elucidate the connection between XIST and asthma, and propose its potential as a biomarker for gender disparities in asthma prevalence. Fagerberg et al. (34) utilized next-generation sequencing to analyze the transcriptomes of 95 different human organs and tissues based on a total of 27 individuals’ samples. They discovered the expression of the XIST gene in human lung tissue. In our analysis of three samples from a cohort of 575 individuals, we observed elevated expression of XIST exclusively in females. Currently, there is a lack of reports regarding the gender differences in XIST expression in the context of asthma. However, the high expression of XIST has been shown to be associated with primary biliary cholangitis in females, XIST can stimulate the proliferation and differentiation of initial CD4^+^ T cells, which considered to be the reason for the high incidence of PBC in females (35). In addition, Yu et al. (36) confirmed that dysregulation of XIST may bias the differentiation selection of this immune cell, with dysregulation of XISL evident in CD11c + atypical B cells in female patients but not in male patients. These results indicate that XIST may affect gender differences in asthma by targeting the proliferation and differentiation of immune cells.

Asthma is associated with sex hormone levels and obesity, and some published researches revealed that XIST is involved in regulating these biological processes. XIST is associated with the expression of sex hormones. Armoskus et al. (37) employed gene expression microarrays to identify 90 potential genes that were differentially expressed in male and female mice’s neocortex/hippocampus, and PCR reverse transcription revealed dimorphic expression of the XIST gene. XIST is implicated in androgen/estrogen signaling pathways, protein modification, and cell proliferation/death, all of which are linked to differences in neurodevelopment, cognitive function, and neurological illness between sexes. Wang et al. (38) discovered that the lncRNA XIST was down-regulated in late-onset hypogonadism, and that XIST siRNA increased cell apoptosis, increased caspase3 activity, and decreased testosterone levels. XIST also regulates obesity-related processes. XIST may assist regulate intramuscular fat metabolism, according to Yang et al. (39), who used bioinformatics analysis and machine learning to uncover potential tissue-specific indicators of swine fat accumulation. Wu et al. (40) discovered that XIST expression was substantially higher in female than male persons in human adipose tissue. XIST expression increased considerably *in vitro* during brown fat cell development. Brown preadipocyte development was impeded by XIST knockdown, but XIST overexpression facilitated full differentiation. Yao et al. (41) used lncrNA-mirNA-mrna networks to identify possible functional lncRNAs in metabolic syndrome (including abdominal obesity), and discovered that XIST was the most relevant lncRNA.

Abnormal proliferation and activation of immune cells are considered to be the key to the pathogenesis of asthma. TH2 cell was generally considered to be the main immune cell responsible for asthma, but increasing evidence shows that asthma was related to B cells (42, 43). Previous research has demonstrated the crucial role of B cells in regulating lung function and airway remodeling in mouse models of asthma (44). Mechanistic investigations have revealed that B cells contribute to the asthmatic process by initiating and sustaining T helper (Th) cell-mediated immune responses (45). A recent study highlighted the connection between the initiation of the Th response and innate lymphoid cells type 2 (ILC2s). ILC2s reside on mucosal surfaces, including the lungs, and are capable of producing type 2 cytokines such as interleukin-5 (IL-5) and interleukin-13 (IL-13), which are pivotal in the pathogenesis of allergic disorders and asthma (46). Notably, IL-13 can induce B cell class switching and the production of immunoglobulin E (IgE), collectively exacerbating the progression of asthma (47). Habener et al. (48) found that IgA + memory B cells were significantly increased in peripheral blood mononuclear cells of asthmatic patients, especially in asthmatic patients with small airway dysfunction. Wypych et al. (45) also confirmed that B cells participate in the pathogenesis of asthma mouse models by amplifying Th cell effects. What is exciting is that the latest study confirmed that XIST was required to maintain the homeostasis of B cells. On the one hand, XIST prevents the escape of x-linked genes with DNA hypomethylation promoters in B cells. On the other hand, XIST maintains X inactivation through sustained deacetylation of H3K27ac, revealing the regulatory role of XIST in B cells (36). Interestingly, XIST dysregulation was found in infiltrating B cells of rheumatoid arthritis joint tissues, which is a chronic inflammatory condition in the same family as asthma (36), suggesting the potential of XIST in the treatment of chronic inflammation, which indirectly justifies the conclusion of the present study that XIST can be used as a therapeutic target for asthma. The study conducted by Zhou et al. (49) provides additional support for our findings. They obtained peripheral blood samples from 137 pediatric asthma patients and 59 healthy children. Through bioinformatics analysis, it was revealed that XIST is significantly upregulated in pediatric asthma patients.

Jiang et al. (50) employed bioinformatics approaches to analyze the hub genes and signaling pathways involved in severe asthma. Through protein–protein interaction network analysis and module analysis, they identified 11 hub genes within key modules. Jiang’s study also involved the GSE76226 dataset, yet it yielded no overlapping results with our predicted genes. We speculate that the probable reason lies in the different methodologies employed: this study utilized machine learning models with parameter optimization techniques to screen for potential genes, whereas Jiang et al. analyzed the top 5,000 genes from three datasets.

We must acknowledge the limitations of this study. Firstly, it is based on predictive analysis of existing databases to identify gender-specific differences in asthma prevalence genes, suggesting XIST as a potential biomarker. However, experimental validation is lacking, and we plan to address this in future experiments. Secondly, our analysis utilized three datasets, with one dataset including age information (over 18 years old), as our preliminary literature review revealed a reversal in asthma prevalence between males and females during adolescence.

## 5 Conclusion

The study, based on machine learning, found genetic biomarkers that caused sex differences in asthma rates around puberty, which has attracted widespread attention. Grid search was used to train and adjust parameters of support vector machine, decision tree, logistic regression and random forest. Results revealed that XIST was a potential genetic biomarker associated with gender differences in asthma prevalence.

## Data Availability

The datasets presented in this study can be found in online repositories. The names of the repository/repositories and accession number(s) can be found at: https://www.ncbi.nlm.nih.gov/geo/, GSE69683, GSE76262 and GSE41863.

## Footnotes

^1^ https://www.ncbi.nlm.nih.gov/geo/
